# Sleep quality, duration, and consistency are associated with better academic performance in college students

**DOI:** 10.1038/s41539-019-0055-z

**Published:** 2019-10-01

**Authors:** Kana Okano, Jakub R. Kaczmarzyk, Neha Dave, John D. E. Gabrieli, Jeffrey C. Grossman

**Affiliations:** 1 MIT Integrated Learning Initiative, Department of Brain and Cognitive Sciences, and McGovern Institute for Brain Research, Massachusetts Institute of Technology, Cambridge, MA 02139 USA; 2 Harvard Business School, Boston, MA 02163 USA; 3 Department of Materials Science and Engineering Massachusetts Institute of Technology, Cambridge, MA 02139 USA

**Keywords:** Education, Education

## Abstract

Although numerous survey studies have reported connections between sleep and cognitive function, there remains a lack of quantitative data using objective measures to directly assess the association between sleep and academic performance. In this study, wearable activity trackers were distributed to 100 students in an introductory college chemistry class (88 of whom completed the study), allowing for multiple sleep measures to be correlated with in-class performance on quizzes and midterm examinations. Overall, better quality, longer duration, and greater consistency of sleep correlated with better grades. However, there was no relation between sleep measures on the single night before a test and test performance; instead, sleep duration and quality for the month and the week before a test correlated with better grades. Sleep measures accounted for nearly 25% of the variance in academic performance. These findings provide quantitative, objective evidence that better quality, longer duration, and greater consistency of sleep are strongly associated with better academic performance in college. Gender differences are discussed.

## Introduction

The relationship between sleep and cognitive function has been a topic of interest for over a century. Well-controlled sleep studies conducted with healthy adults have shown that better sleep is associated with a myriad of superior cognitive functions,^1–6^ including better learning and memory.^7,8^ These effects have been found to extend beyond the laboratory setting such that self-reported sleep measures from students in the comfort of their own homes have also been found to be associated with academic performance.^9–13^

Sleep is thought to play a crucial and specific role in memory consolidation. Although the exact mechanisms behind the relationship between sleep, memory, and neuro-plasticity are yet unknown, the general understanding is that specific synaptic connections that were active during awake-periods are strengthened during sleep, allowing for the consolidation of memory, and synaptic connections that were inactive are weakened.^5,14,15^ Thus, sleep provides an essential function for memory consolidation (allowing us to remember what has been studied), which in turn is critical for successful academic performance.

Beyond the effects of sleep on memory consolidation, lack of sleep has been linked to poor attention and cognition. Well-controlled sleep deprivation studies have shown that lack of sleep not only increases fatigue and sleepiness but also worsens cognitive performance.^2,3,16,17^ In fact, the cognitive performance of an individual who has been awake for 17 h is equivalent to that exhibited by one who has a blood alcohol concentration of 0.05%.^1^ Outside of a laboratory setting, studies examining sleep in the comfort of peoples’ own homes via self-report surveys have found that persistently poor sleepers experience significantly more daytime difficulties in regards to fatigue, sleepiness, and poor cognition compared with persistently good sleepers.^18^

Generally, sleep is associated with academic performance in school. Sleep deficit has been associated with lack of concentration and attention during class.^19^ While a few studies report null effects,^20,21^ most studies looking at the effects of sleep quality and duration on academic performance have linked longer and better-quality sleep with better academic performance such as school grades and study effort.^4,6,9–13,22–27^ Similarly, sleep inconsistency plays a part in academic performance. Sleep inconsistency (sometimes called “social jet lag”) is defined by inconsistency in sleep schedule and/or duration from day to day. It is typically seen in the form of sleep debt during weekdays followed by oversleep on weekends. Sleep inconsistency tends to be greatest in adolescents and young adults who stay up late but are constrained by strict morning schedules. Adolescents who experience greater sleep inconsistency perform worse in school.^28–31^

Although numerous studies have investigated the relationship between sleep and students’ academic performance, these studies utilized subjective measures of sleep duration and/or quality, typically in the form of self-report surveys; very few to date have used objective measures to quantify sleep duration and quality in students. One exception is a pair of linked studies that examined short-term benefits of sleep on academic performance in college. Students were incentivized with offers of extra credit if they averaged eight or more hours of sleep during final exams week in a psychology class^32^ or five days leading up to the completion of a graphics studio final assignment.^33^ Students who averaged eight or more hours of sleep, as measured by a wearable activity tracker, performed significantly better on their final psychology exams than students who chose not to participate or who slept less than eight hours. In contrast, for the graphics studio final assignments no difference was found in performance between students who averaged eight or more hours of sleep and those who did not get as much sleep, although sleep consistency in that case was found to be a factor.

Our aim in this study was to explore how sleep affects university students’ academic performance by objectively and ecologically tracking their sleep throughout an entire semester using Fitbit—a wearable activity tracker. Fitbit uses a combination of the wearer’s movement and heart-rate patterns to estimate the duration and quality of sleep. For instance, to determine sleep duration, the device measures the time in which the wearer has not moved, in combination with signature sleep movements such as rolling over. To determine sleep quality, the Fitbit device measures the wearer’s heart-rate variability which fluctuates during transitions between different stages of sleep. Although the specific algorithms that calculate these values are proprietary to Fitbit, they have been found to accurately estimate sleep duration and quality in normal adult sleepers without the use of research-grade sleep staging equipment.^34^ By collecting quantitative sleep data over the course of the semester on nearly 100 students, we aimed to relate objective measures of sleep duration, quality, and consistency to academic performance from test to test and overall in the context of a real, large university college course.

A secondary aim was to understand gender differences in sleep and academic performance. Women outperform men in collegiate academic performance in most subjects^35–38^ and even in online college courses.^39^ Most of the research conducted to understand this female advantage in school grades has examined gender differences in self-discipline,^40–42^ and none to date have considered gender differences in sleep as a mediating factor on school grades. There are inconsistencies in the literature on gender differences in sleep in young adults. While some studies report that females get more quantity^43^ but worse quality sleep compared with males,^43,44^ other studies report that females get better quality sleep.^45,46^ In the current study, we aim to see whether we would observe a female advantage in grades and clarify how sleep contributes to gender differences.

## Results

### Bedtime and wake-up times

On average, students went to bed at 1:54 a.m. (Median = 1:47 a.m., Standard Deviation (SD) of all bedtime samples = 2 h 11 min, SD of mean bedtime per participant = 1 h) and woke up at 9:17 a.m. (Median = 9:12 a.m., SD of all wake-up time samples = 2 h 2 min; SD of mean wake-up time per participant = 54 min). The data were confirmed to have Gaussian distribution using the Shapiro–Wilks normality test. We conducted an ANOVA with the overall score (sum of all grade-relevant quizzes and exams—see “Procedure”) as the dependent variable and bedtime (before or after median) and wake-up time (before or after median) as the independent variables. We found a main effect of bedtime (*F* (1, 82) = 6.45, *p* = 0.01), such that participants who went to bed before median bedtime had significantly higher overall score (*X* = 77.25%, SD = 13.71%) compared with participants who went to bed after median bedtime (*X* = 70.68%, SD = 11.01%). We also found a main effect of wake-up time (*F* (1, 82) = 6.43, *p* = 0.01), such that participants who woke up before median wake-up time had significantly higher overall score (*X* = 78.28%, SD = 9.33%) compared with participants who woke up after median wake-up time (*X* = 69.63%, SD = 14.38%), but found no interaction between bedtime and wake-up time (*F* (1, 82) = 0.66, *p* = 0.42).

A Pearson’s product-moment correlation between average bedtime and overall score revealed a significant and negative correlation (*r* (86) = −0.45, *p* < 0.0001), such that earlier average bedtime was associated with a higher overall score. There was a significant and negative correlation between average wake-up time and overall score (*r* (86) = −0.35, *p* < 0.001), such that earlier average wake-up time was associated with a higher overall score. There was also a significant and positive correlation between average bedtime and average wake-up time (r (86) = 0.68, *p* < 0.0001), such that students who went to bed earlier tended to also wake up earlier.

### Sleep duration, quality, and consistency in relation to academic performance

**Fig. 1:**
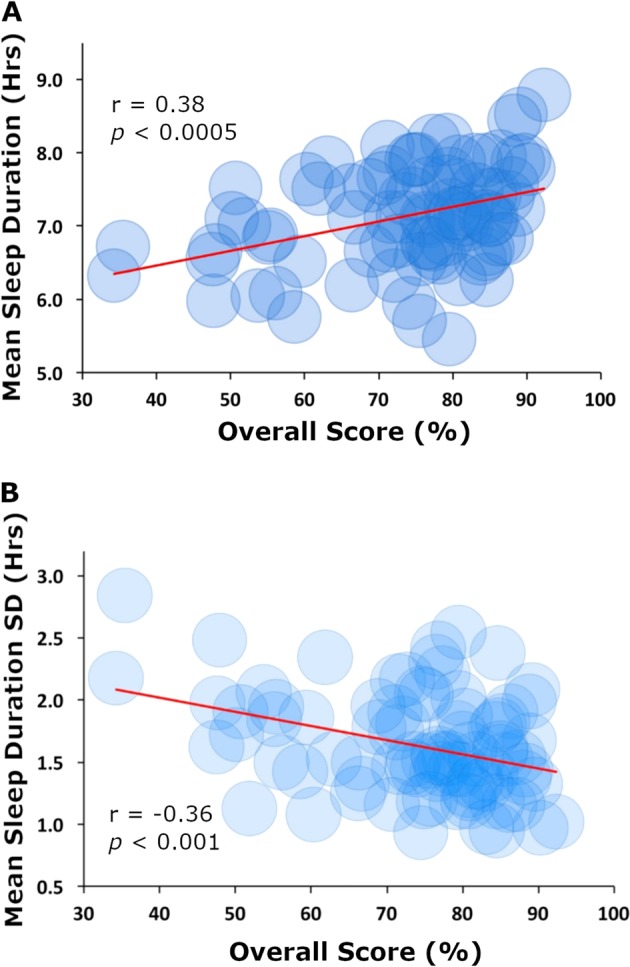
Correlations between sleep measures and overall score. **a** Average daily hours slept (sleep duration) vs. overall score for the semester. **b** Standard deviation of average daily hours of sleep (sleep inconsistency) vs. overall score in class

Overall, the mean duration of sleep for participants throughout the entire semester was 7 h 8 min (SD of all sleep samples = 1 h 48 min, SD of mean sleep duration per participant = 41 min). There was a significant positive correlation between mean sleep duration throughout the semester (sleep duration) and overall score (*r* (86) = 0.38, *p* < 0.0005), indicating that a greater amount of sleep was associated with a higher overall score (Fig. 1a). Similarly, there was a significant positive correlation between mean sleep quality throughout the semester (Sleep Quality) and Overall Score (*r* (86) = 0.44, *p* < 0.00005). Sleep inconsistency was defined for each participant as the standard deviation of the participant’s daily sleep duration in minutes so that a larger standard deviation indicated greater sleep inconsistency. There was a significant negative correlation between sleep inconsistency and overall score (*r* (86) = −0.36, *p* *<* 0.001), indicating that the greater inconsistency in sleep duration was associated with a lower overall score (Fig. 1b).

### Timing of sleep and its relation to academic performance

To understand sleep and its potential role in memory consolidation, we examined the timing of sleep as it related to specific assessments. All Pearson correlations with three or more comparisons were corrected for multiple comparisons using false discovery rate.^47^

#### Night before assessments

We conducted a correlation between sleep quality the night before a midterm and respective midterm scores as well as sleep duration the night before a midterm and respective scores. There were no significant correlations with sleep duration or sleep quality for all three midterms (all *r*s < 0.20, all *p*s > 0.05). Similar analyses for sleep duration and sleep quality the night before respective quizzes revealed no correlations (*r*s from 0.01 to 0.26, all *p*s > 0.05).

#### Week and month leading up to assessments

To understand the effect of sleep across the time period while course content was learned for an assessment, we examined average sleep measures during the 1 month leading up to the midterms. We found a significant positive correlation between average sleep duration over the month leading up to scores on each midterm (*r*s from 0.25 to 0.34, all *p*s < 0.02). Similar analyses for average sleep duration over one week leading up to respective quizzes were largely consistent with those of midterms, with significant correlations on 3 of 8 quizzes (rs from 0.3 to 0.4, all *p*s < 0.05) and marginal correlations on an additional 3 quizzes (rs from 0.25 to 0.27, all *p*s < 0.08).

There was a significant and positive correlation between sleep quality scores averaged over the month leading up to each midterm for all three midterms (*r*s from 0.21 to 0.38, all *p*s < 0.05). Similar analyses for average Sleep Quality over one week leading up to respective quizzes revealed a significant correlation on 1 of 8 quizzes (*r* (86) = 0.42, *p* < 0.005) and marginal correlations on 3 quizzes (*r*s from 0.25 to 0.27, all *p*s < 0.08).

### Variance of assessment performance accounted for by sleep measures

In order to calculate how much of the variance on assessment performance was accounted for by the sleep measures, we conducted a stepwise regression on overall score using three regressors: sleep duration, sleep quality, and sleep inconsistency. The relative importance of each variable was calculated using the relaimpo package in *R*^48^ to understand individual regressor’s contribution to the model, which is not always clear from the breakdown of model *R*^2^ when regressors are correlated. We found a significant regression (*F* (3,84) = 8.95, *p* = .00003), with an *R*^2^ of 0.24. Students’ predicted overall score was equal to 77.48 + 0.21 (sleep duration) + 19.59 (Sleep Quality) – 0.45 (sleep inconsistency). While sleep inconsistency was the only significant individual predictor of overall score (*p* = 0.03) in this analysis, we found that 24.44% of variance was explained by the three regressors. The relative importance of these metrics were 7.16% sleep duration, 9.68% sleep quality, and 7.6% sleep inconsistency.

### Gender differences

Females had better Sleep Quality (*t* (88) = 2.63, *p* = 0.01), and less sleep inconsistency (*t* (88) = 2.18, *p* = 0.03) throughout the semester compared with males, but the two groups experienced no significant difference in sleep duration (*t* (88) = 1.03, *p* = 0.3). Sleep duration and sleep quality were significantly correlated in both males (*r* (41) = 0.85, *p* < 0.00001) and females (*r* (43) = 0.64, *p* < 0.00001), but this correlation was stronger in males (*Z* = −2.25, *p* = 0.02) suggesting that it may be more important for males to get a long-duration sleep in order to get good quality sleep. In addition, sleep inconsistency and sleep quality were significantly negatively correlated in males (*r* (41) = −0.51, *p* = 0.0005) but not in females (*r* (43) = 0.29, *p* > 0.05), suggesting that it may be more important for males to stick to a regular daily sleep schedule in order to get good quality sleep.

Females scored higher on overall score compared with males (*t* (88) = −2.48, *p* = 0.01), but a one-way analysis of covariance (ANCOVA) revealed that females and males did not perform significantly different on overall score when controlling for Sleep Quality, *F* (1, 85) = 2.22, *p* = 0.14. Sleep inconsistency and overall score were negatively correlated in males (*r* (41) = −0.44, *p* = 0.003) but not in females (*r* (43) = −0.13, *p* = 0.39), suggesting that it is important for males to stick to a regular sleep schedule in order to perform well in academic performance but less so for females. No other gender differences were detected between other sleep measures and overall score.

## Discussion

This study found that longer sleep duration, better sleep quality, and greater sleep consistency were associated with better academic performance. A multiple linear regression revealed that these three sleep measures accounted for 24.44% of the variance in overall grade performance. Thus, there was a substantial association between sleep and academic performance. The present results correlating overall sleep quality and duration with academic performance are well aligned with previous studies^6,11,12,24,25^ on the role of sleep on cognitive performance. Similarly, this study compliments the two linked studies that found longer sleep duration during the week before final exams^47^ and consistent sleep duration five days prior to a final assignment^48^ enhanced students’ performance. The present study, however, significantly extends our understanding of the relation between sleep and academic performance by use of multiple objective measures of sleep throughout an entire semester and academic assessments completed along the way.

The present study also provides new insights about the timing of the relation between sleep and academic performance. Unlike a prior study,^23^ we did not find that sleep duration the night before an exam was associated with better test performance. Instead we found that both longer sleep duration and better sleep quality over the full month before a midterm were more associated with better test performance. Rather than the night before a quiz or exam, it may be more important to sleep well for the duration of the time when the topics tested were taught. The implications of these findings are that, at least in the context of an academic assessment, the role of sleep is crucial during the time the content itself is learned, and simply getting good sleep the night before may not be as helpful. The outcome that better “content-relevant sleep” leads to improved performance is supported by previous controlled studies on the role of sleep in memory consolidation.^14,15^

Consistent with some previous research^45,46^ female students tended to experience better quality sleep and with more consistency than male students. In addition, we found that males required a longer and more regular daily sleep schedule in order to get good quality sleep. This female advantage in academic performance was eliminated once sleep patterns were statistically equated, suggesting that it may be especially important to encourage better sleep habits in male students (although such habits may be helpful for all students).

Several limitations of the present study may be noted. First, the sleep quality measures were made with proprietary algorithms. There is an evidence that the use of cardiac, respiratory, and movement information from Fitbit devices can accurately estimate sleep stages,^32^ but there is no published evidence that Fitbit’s 1~10 sleep quality scores represent a valid assessment of sleep quality. Second, the relation between sleep and academic performance may be moderated by factors that can affect sleep, such as stress, anxiety, motivation, personality traits, and gender roles. Establishing a causal relation between sleep and academic performance will require experimental manipulations in randomized controlled trials, but these will be challenging to conduct in the context of real education in which students care about their grades. Third, these findings occurred for a particular student population at MIT enrolled in a particular course, and future studies will need to examine the generalizability of these findings to other types of student populations and other kinds of classes.

In sum, this study provides evidence for a strong relation between sleep and academic performance using a quantifiable and objective measures of sleep quality, duration, and consistency in the ecological context of a live classroom. Sleep quality, duration, and consistency together accounted for a substantial amount (about a quarter) of the overall variance in academic performance.

## Methods

### Participants

One hundred volunteers (47 females) were selected from a subset of students who volunteered among 370 students enrolled in Introduction to Solid State Chemistry at the Massachusetts Institute of Technology to participate in the study. Participants were informed of the study and gave written consent obtained in accordance with the guidelines of and approved by the MIT Committee on the Use of Humans as Experimental Subjects. Due to limitations in funding, we only had access to 100 Fitbit devices and could not enroll all students who volunteered; consequently, the first 100 participants to volunteer were selected. All participants were gifted a wearable activity tracker at the completion of the study in exchange for their participation. Seven participants were excluded from analysis because they failed to wear their activity tracker for more than 80% of the semester, three participants were excluded because they lost their wearable activity tracker, and another two participants were excluded because they completed less than 75% of the assessments in the class. Of the 88 participants who completed the study (45 females), 85 were freshmen, one was a junior and two were seniors (mean age = 18.19 years).

The Solid State Chemistry class is a single-semester class offered in the fall semester and geared toward freshmen students to satisfy MIT’s general chemistry requirement. The class consisted of weekly lectures by the professor and two weekly recitations led by 12 different teaching assistants (TAs). Each student was assigned to a specific recitation section that fit their schedule and was not allowed to attend other sections; therefore, each student had the same TA throughout the semester. Students took (1) weekly quizzes that tested knowledge on the content covered the week leading up to the quiz date, (2) three midterms that tested knowledge on the content covered in the 3–4 weeks leading up to the exam date, and (3) a final exam that tested content covered throughout the semester. Based on a one-way between subjects’ analysis of variance (ANOVA) to compare the effect of teaching assistants (TAs) on overall grade, we found no significant differences in overall grade across the TAs (F (10, 77) = 1.82, *p* = 0.07. (One TA was removed from the analysis because he only had one student who was participating in this study).

### Procedure

Participants were asked to wear an activity tracker for the entire duration of the semester without going below 80% usage each week. If 80% or more usage was not maintained, warning emails were sent at the end of that respective week. Participants were asked to return the device if they dipped below 80% usage more than three out of the 14 weeks of the semester. The average usage rate at the end of the semester for the 88 participants who completed the study was 89.4% (SD = 5.5%). The missing data appeared to be at random and were deleted prior to data analysis. As part of a separate research question, 22 of the 88 participants joined an intense cardiovascular exercise class for which they received separate physical education credit. These students performed similarly to the other 67 participants in terms of final class grade (*t* (88) = 1.57, *p* = 0.12), exercise amount (total amount of moderately and very active minutes on the wearable device) (t (88) = 0.59, *p* = 0.56), sleep amount (*t* (88) = 0.3, *p* = 0.77), and sleep quality (*t* (88) = 0.14, *p* = 0.9), so they were included in all of the analyses.

### Materials

Participants’ activities were tracked using a Fitbit Charge HR. Data from the device were recorded as follows: heart rate every 5 min; steps taken, distance traveled, floors climbed, calories burned and activity level measurements every 15 min; resting heart rate daily; and sleep duration and quality for every instance of sleep throughout the day. Sleep quality was determined using Fitbit’s proprietary algorithm that produces a value from 0 (poor quality) to 10 (good quality).

### Assessments

Nine quizzes, three midterm examinations, and one final examination were administered throughout the 14-week class to assess the students’ academic achievement. The students’ cumulative class grade was made up of 25% for all nine quizzes (lowest quiz grade was dropped from the average), 15% for each midterm exam, and 30% for the final exam for a total of 100%.

At MIT, freshmen are graded on a Pass or No Record basis in all classes taken during their first semester. Therefore, all freshmen in this class needed a C- level or better (≥50%, no grading on a curve) to pass the class. A failing grade (a D or F grade) did not go on their academic record. All upperclassmen were given letter grades; A (≥85%), B (70–84%), C (50–69%), D (45–49%), F (≤44%). Because a large portion of the class had already effectively “passed” the class before taking Quiz 9 and the final exam, we excluded these two assessments from our analyses due to concerns about students’ motivation to perform their best. We calculated for each student an overall score defined as the sum of the eight quizzes and three midterms to summarize academic performance in the course.

### Reporting summary

Further information on research design is available in the Nature Research Reporting Summary linked to this article.

## Supplementary information

Reporting Summary

## Data Availability

The data that support the findings of this study are available from the corresponding author upon reasonable request.

## Supplementary information

**Supplementary Information** accompanies the paper on the *npj Science of Learning* website (10.1038/s41539-019-0055-z).

## Code availability

No custom codes were used in the analysis of this study
